# Overview and outcome of Hodgkin’s Lymphoma: Experience of a single developing country’s oncology centre

**DOI:** 10.1371/journal.pone.0195629

**Published:** 2018-04-12

**Authors:** Rawand P. Shamoon, Mohamad Dahir Ali, Nazar P. Shabila

**Affiliations:** 1 Department of Pathology, College of Medicine, Hawler Medical University, Erbil, Iraq; 2 Department of Haematopathology, Nanakali Haemato-Oncology Teaching Centre, Erbil, Iraq; 3 Department of Clinical Haematology, Nanakali Hemato-Oncology Teaching Centre, Erbil, Iraq; 4 Department of Community Medicine, Hawler Medical University, Erbil, Iraq; European Institute of Oncology, ITALY

## Abstract

Hodgkin’s Lymphoma (HL) reveals variable epidemiological and clinico-pathological features in different geographical locations. In this retrospective study, we aimed to assess the epidemiological and clinic-pathological features, and outcome of HL patients treated at one hemato-oncology centre in Erbil, northern Iraq. Medical records of 103 HL patients treated over more than six years were reviewed. Treatment outcome was evaluated by measuring the 5-year overall and progression-free survival rates. The median age of patients was 23 years, children up to 17 years constituted 31.1%, and male to female ratio was 1:1.05. The majority (96.1%) of patients presented with lymphadenopathy. Nodular sclerosis subtype was the mostly encountered histologic type (48.5%); about half of the patients (49.5%) had stage II disease. Relapse occurred in 20 patients; the 5-year overall survival for children was better (89%) compared to adult patients (79%). The associated risk features found to have adverse effects on the survival, however, only high LDH level and presence of B-symptoms at presentation showed significant correlation. The epidemiological and clinical characteristics of HL in our locality followed the pattern in the western world. The 5-year overall and progression-free survivals were far below the international rates, a matter which may necessitate a revision to HL treatment strategy at our centre.

## Introduction

Hodgkin Lymphoma (HL) is an uncommon tumour, although it is one of the more frequent malignancies in young people. Its annual incidence is 2–3 per 100,000 in Europe and the USA; though it accounts for 5–6% of all childhood cancer. There are variations in the epidemiologic and clinico-pathological characteristics of HL in relation to geography and socioeconomic status. In the industrialised countries, HL has a bimodal incidence with the main peak in young adults of 15–35 years and the second one occurring after the age of 50. On the other hand, the disease appears more in young children in the developing countries. Males are affected more often than females in all subtypes; however, the nodular sclerosing type occurs slightly more often in young females [1,2].

HL is considered as one of the malignant diseases that respond well to treatment. With continuing clinical trials and combination therapy over the last 30 years, survival rates have been continuously raised. The improvement in patients’ survival is mainly remarkable in the paediatric age and low risk groups [3–6]. Fortunately, the success story of HL is not restricted to the developed world; promising rates of survival have been reported from many developing areas [7–11]. Reducing short and long-term toxicity with maintaining excellent cure rates has become the principal objective of the recent trials in the developed part of the world [12,13]. This attempt may be difficult in our setting where patients, as well as physicians, often choose certainty of cure over the risk of late effect.

In our centres, and like the case in some other centres in the developing world, HL patients are principally treated with extensive cycles of ABVD (Doxorubicin, Bleomycin, Vinblastine, and Dacarbazine) chemotherapy. There are increased reports of promising survival rates of HL (80–95%) in the last two decades [14–16]. However, there is lack of outcome data about HL patients who have been treated at our centre. Therefore, we deemed necessary to review and study our records to assess the quality of care and the effectiveness of the treatment protocols applied at our centre. Hence, this study aimed to describe the demographic and clinico-pathological aspects of patients with HL, assess the 5-year overall (OS) and progression-free survival (PFS) rates of paediatric and adult patients, and evaluate the effect of the associated unfavourable risk factors on patients’ survival.

## Patients and methods

This retrospective study was carried out at Nanakali Hemato-Oncology Teaching Centre in Erbil. Nanakali Centre is a 100-bed public hospital that receives paediatric and adult patients with benign and malignant haematology diseases and solid tumours. Monthly, an average of 120 new and/or follow up cases are admitted and treated for free at this centre.

Records of 103 patients (32 children/adolescents, 71 adults) with HL who were registered and treated at Nanakali Centre from May 2010 to December 2016 were reviewed. Eighteen patients were excluded from this analysis; eight have escaped the follow up and ten have chosen to be treated outside the country after they were diagnosed. The included patients were fully anonymised before accessing their files and the study was approved by the ethical committee of the Hawler Medical University. Demographic data, presenting symptoms, examination findings, mainly number and size of nodal and extranodal regions, routine laboratory and histopathology results, treatment plan, and treatment outcome were retrieved from patients’ records. The diagnosis of HL was made based on routine histopathologic and immunophenotypic studies done at the histopathology referral lab in Rizgary Teaching Hospital, Erbil. Cases were histologically classified according to the WHO classification [17]. At diagnosis, all patients had chest radiography, abdominal ultrasonography, as well as computed tomography (CT) scans of neck, chest, and abdomen. Some patients (24/103) were evaluated by fluorodeoxyglucose-positron emission tomography (FDG-PET) scan. FDG-PET was available for a limited number of patients who could afford it outside Iraq. Bone marrow aspiration and biopsy were performed for all patients. Clinical staging followed the Ann Arbor classification [18] and was decided upon by the hemato-oncology committee at Nanakali Centre based on the clinical and radiological findings as well as the results of laboratory investigations. Bulky disease was defined as lymph node (LN) mass ≥10cm diameter or mediastinal mass exceeding one-third of the maximum mediastinal width on an upright posteroanterior chest radiograph. Patients with stages I and II-A were considered as early stages and those with stages II-B, III, and IV were considered as advanced stages of the disease.

### Treatment protocol

HL patients were treated only with ABVD chemotherapy. Involved field radiotherapy (dose range: 25-30Gy, over 12–15 days) was used only for adult patients with bulky disease. A varying number of ABVD cycles were used according to the stage of disease and/or presence of associated risk features.

### Children and adolescents

Patients with localised disease (stage I and II-A) were treated with four cycles ABVD. Patients with stage II-B and III diseases were treated with six cycles ABVD. Patients with stage IV disease were treated with eight cycles ABVD. Patients with the relapsed disease were treated with four cycles COPP-ABV (cyclophosphamide, vincristine, prednisolone, procarbazine, doxorubicin, bleomycin, vinblastine) protocol.

### Adults

Patients with localised disease (stage I and II-A) were treated with four cycles ABVD. Patients with stage II disease who had B-symptoms and/or one or more of the following unfavourable features at presentation: mediastinal mass >10cm, extranodal disease, and involvement of >3 nodal sites were given six cycles ABVD therapy. Patients with advanced clinical stage (stage III and IV) were treated with eight cycles ABVD.

The treatment was started with administering two to three cycles of ABVD chemotherapy, and then patients were re-examined to evaluate the response. Response to therapy was evaluated depending on clinical judgment and CT scan (or FDG-PET in some) results. Good responders, defined as diminished clinical symptoms and tumour size regression to >50% of its initial size, continued to complete their chemotherapy plan as per their clinical stages. Refractory cases with poor response, defined as persistence of clinical symptoms and/or minimal reduction of the initial tumour size, were switched to 4-cycle ICE (Ifosfamide, Carboplatin, Etoposide) or DHAP (Dexamethasone, high-dose Cytarabine, Cisplatin) chemotherapy protocols. Cases with the relapsed disease were treated with 4-cycle ICE or DHAP protocols as well.

The treatment outcome was evaluated by measuring the 5-year OS and PFS rates. The associated risk features that could be relevant to survival and that were evaluated in relation to the prognosis included age, gender, histologic type, site of the disease, stage, the presence of B-symptoms, ESR, and serum LDH level.

### Statistical analysis

The data were analysed using the statistical package for the social sciences (version 19). The estimates of OS and PFS were calculated using the life table method. Kaplan–Meier method and the log–rank test were used to estimate the differences in OS and PFS among the patients. A P value of ≤0.05 was considered statistically significant for all statistical tests. All the variables mentioned above were included as covariates in the multivariate analysis using Cox regression model. Hazard ratios and the corresponding 95% confidence intervals (CI) were constructed in models adjusted for all listed covariates of interest.

## Results

During the period between May 2010 and December 2016, 103 patients with HL were diagnosed, admitted, and treated at Nanakali Hemato-oncology Teaching Centre in Erbil. The age of diagnosis ranged from 3 to 83 years with a median of 23 years; children between 3 and 17 years constituted 31.1% (32 cases). Fifty patients (48.5%) were male; male to female ratio was 1:1.05. Among children, 17 patients (53%) were male and 15 (47%) were female.

In the current cohort, the majority (n = 99, 96.1%) of the patients presented with lymphadenopathy. The most common nodal sites involved were cervical and mediastinal (n = 73, 70.9% and n = 10, 9.7%, respectively). Four patients had an extranodal disease at presentation, two of which with pulmonary involvement. B-symptoms were encountered in 61 patients. Concerning the histologic types of the disease, nodular sclerosis (NS) was observed to be the predominant type, affecting 50 patients (48.5%), followed by mixed cellularity (MC) in 47 patients (45.6%). All patients were staged using the Ann Arbor staging system. Seventy patients (68%) had localised disease (stage I/II) and 33 others (32%) had by definition Ann Arbor stage III/IV disease (Table 1).

**Table 1:** Demographic and clinico-pathological characteristics of patients with HL.

Characteristic		N (= 103)	%
**Age (yrs)**	0–17	32	31.1
	18+	71	68.9
**Gender**	Male	50	48.5
	Female	53	51.5
**Histologic type**	NLP	3	2.9
	NS	50	48.5
	MC	47	45.6
	LR	1	1
	LD	2	1.9
**Primary site**	Peripheral LNs	81	78.6
	Deep LNs/ Internal organs	22	21.4
**Stage**	I	19	18.5
	II	51	49.5
	III	27	26.2
	IV	6	5.8
**B-symptoms**	Yes	61	59.2
	No	42	40.8
**ESR (mm/hr)**	≤50	53	51.5
	>50	50	48.5
**Serum LDH (IU/L)**	<500	69	67
	≥500	34	33
**Treatment**	4 ABVD cycles	26	25.2
	4 ABVD cycles + R	9	8.7
	6 ABVD cycles	50	48.5
	6 ABVD cycles + R	8	7.8
	8 ABVD cycles	7	6.8
	8 ABVD cycles + R	3	2.9
**Outcome**	Complete remission	79	76.7
	Relapse	20	19.4
	Death	4	3.9

R: Involved field radiotherapy (for bulky disease); NLP: Nodular lymphocyte predominant; NS: Nodular sclerosis; MC: Mixed cellularity; LR: Lymphocyte-rich; LD: Lymphocyte-depletion

ABVD: Doxorubicin, Bleomycin, Vinblastine, Dacarbazine

The mean period of following up the patients was 24.4 months. A total of 99 patients achieved remission and are still alive, while four patients (3.88%) had died. Of patients who achieved remission, 20 patients (19.4%) had a relapse, were treated, and are still alive. The majority of relapses (18/20) occurred within the first 24 months of diagnosis.

**Fig 1:**
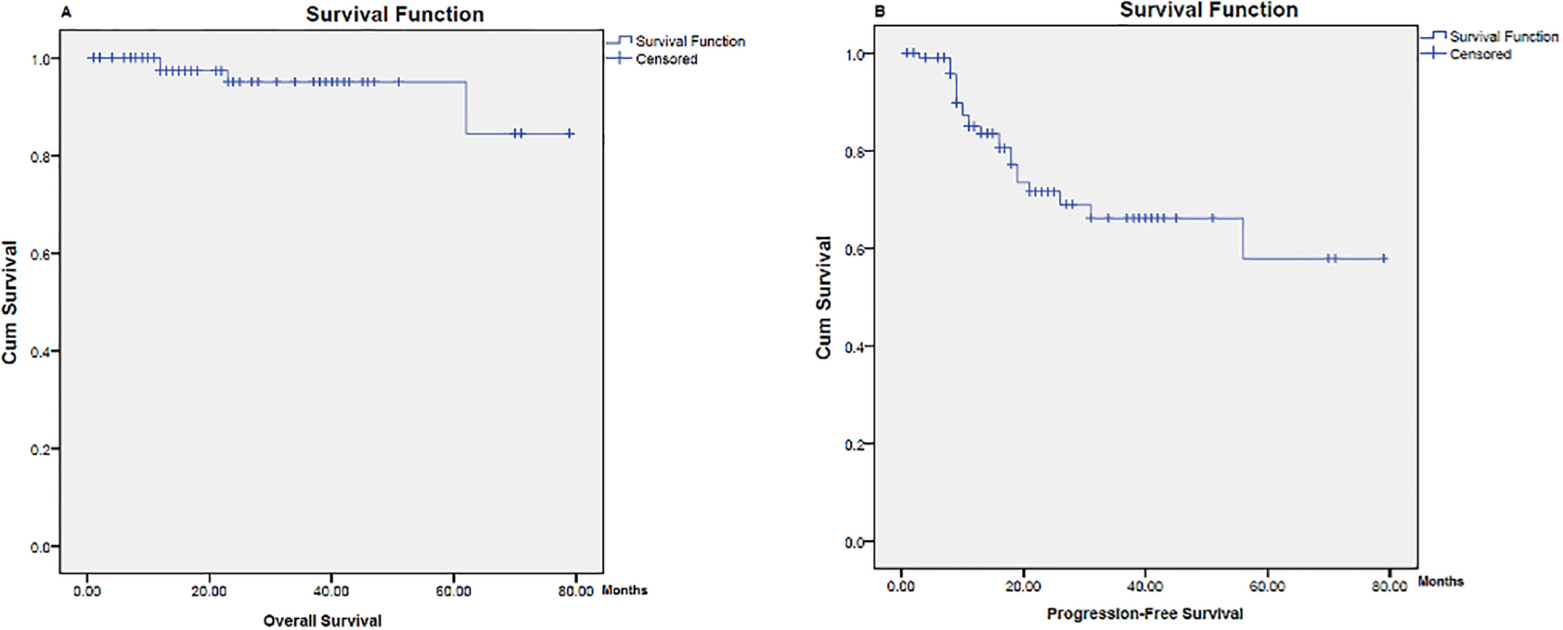
Five-year overall and progression-free survival rates of Hodgkin Lymphoma patients. (A) Five-year Overall Survival Rate. (B) Five-year Progression Survival Rate.

**Fig 2:**
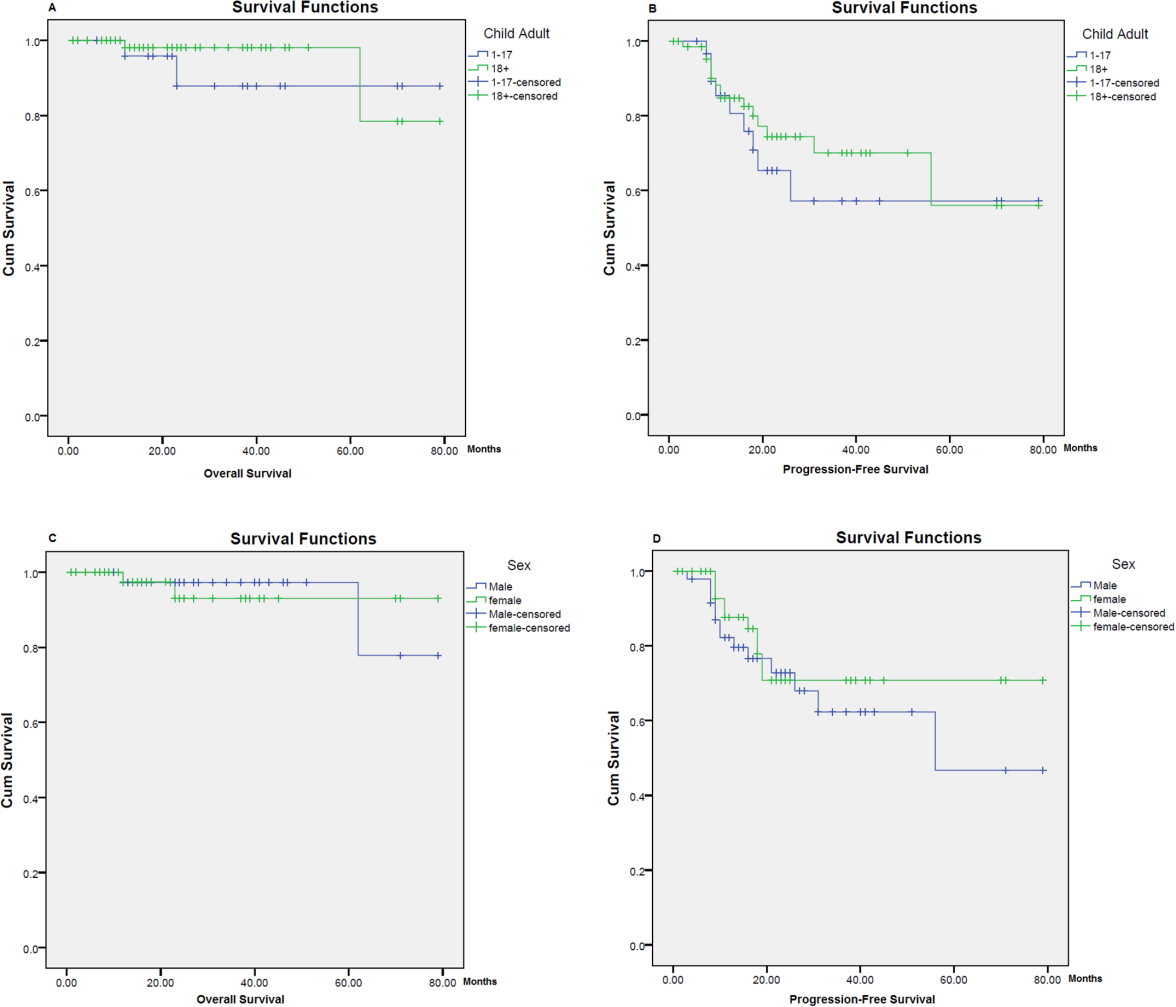
Five-year overall and progression-free survival rates in relation to age and gender. (A) Five-year Overall Survival Rate in Relation to Age. (B) Five-year Progression Survival Rate in Relation to Age. (C) Five-year Overall Survival Rate in Relation to Gender. (D) Five-year Progression Survival Rate in Relation to Gender.

The 5-year overall survival (OS) and progression free survival (PFS) rates of our study were 79% and 60%, respectively (Fig 1A and 1B). The effect of the associated clinical and pathological risk features of the disease on the OS and PFS rates were elaborated by univariable and multivariable analysis. Univariable analysis showed that none of the associated risk features had any significant correlation with the 5-year OS; though, the 5-year PFS showed significant association with both high serum LDH level and presence of B-symptoms at time of presentation (P-values = 0.001 and 0.03 respectively) (Fig 2A–2D; Table 2). On multivariate analysis, only high LDH level revealed a significant association with the 5-year PFS (hazard ratio = 3.655, 95.0% CI 1.58–8.43) (Table 3).

**Table 2:** Association between overall survival and progression-free survival rates and the associated risk features.

Characteristics	Five-year OS		Five-year PFS	
	Rate (%)	P	Rate (%)	P
**Gender**				
**Male**	69	0.996	49	0.380
**Female**	93		72	
**Age**				
**0–17**	89	0.496	56	0.526
**18+**	70		59	
**Primary site**				
**Peripheral**	95	0.149	69	0.271
**Deep**	45		41	
**B-Symptoms**				
**Yes**	61	0.42	15	0.03
**No**	42		5	
**Stage**				
**I, II**	95	0.672	66	0.825
**III, IV**	68		57	
**ESR (mm/hr)**				
**≤50**	96	0.634	59	0.197
**>50**	77		63	
**LDH level (U/L)**				
**<500**	95	0.327	78	0.001
**≥500**	47		26	

**Table 3:** Multivariate analysis of risk features for overall and progression-free survival rates.

	OS				PFS			
	Sig.	Hazard ratio	95.0% CI for Hazard ratio		Sig.	Hazard ratio	95.0% CI for Hazard ratio	
			Lower	Upper			Lower	Upper
**Sex (Female)**	0.767	0.712	0.076	6.711	0.360	0.663	0.275	1.599
**Age (Adult)**	0.232	0.208	0.016	2.731	0.204	0.554	0.223	1.378
**Stage (III & IV)**	0.770	0.653	0.038	11.344	0.206	0.528	0.196	1.421
**Site (internal)**	0.184	5.429	0.447	66.010	0.387	1.544	0.577	4.130
**LDH (≥500)**	0.373	2.691	0.305	23.762	0.002	3.655	1.584	8.435
**ESR (>50)**	0.639	1.748	0.169	18.042	0.204	0.590	0.261	1.332
**B-symptoms (Yes)**	0.747	1.691	0.070	41.096	0.135	2.296	0.773	6.824

## Discussion

In the current retrospective study, we have described the clinical and pathological characteristics as well as the treatment outcome of 103 patients with HL who have been treated at Nanakali Hemato-oncology Centre in Iraqi Kurdistan. Children and adolescents constituted less than one-third (32 patients, 31.1%) of the studied patients. Male to female ratio was 1:1.05; this is contrary to many reports which showed male predominance in HL mainly in low socioeconomic communities [19,20].

The WHO classifies HL into the classical HL (~95% of cases) and the NLP HL (~5% of cases); the former includes the histology subtypes NS, MC, LR, and LD; whereas NLP HL is regarded as a distinctive type compared with classical HL [21]. The majority of our patients (n = 100, 97%) had the classical HL with NS subtype being the most common (48.5%), followed by MC subtype (45.6%). The overall age, sex, and histological distribution of our HL cases is more approximate to the pattern in the western countries and is fairly different to the usual picture in the developing world, where the MC type is reported to be the predominant type, particularly in males [8,11,22,23]. Many studies in Europe have reported NS as the most predominant subtype regardless of age [20,24–26]. Interestingly, a recent study from the nearby city of Mosul, northern Iraq, reported 78.6% NS histology type HL [27]. These findings may indicate that the etiological role of Epstein-Bar virus (EBV) infection in the pathogenesis of HL, which is well established in the developing and poor socioeconomic communities, is not of that extent in our geographical location [28–30].

More than two-thirds of our patients (70 patients, 68%) had early stage disease (stage I and II) at the diagnosis with stage II clinical stage being the most frequently encountered (51 patients, 49.5%). Similarly, the Surveillance, Epidemiology, and End Results program (SEER), which analysed data of 21,734 HL in the United States, reported figures of 19%, 49%, 19%, and 13% for clinical stages I, II, III, and IV, respectively [31]. Other studies from Europe have reported similar figures [7,32]. In contrary, at least more than half of HL patients in developing countries are diagnosed with advanced stage disease [9,10,33,34].

The survival figures of the current cohort are relatively low. The 5-year OS rate was 79%, which is considerably lower in comparison to the survival figures of many regions, including the developing countries [35]. In the current study, the OS in children and adolescents was 89%; which is slightly lower than the international rates [8,11,26,36]. Though, the results were discouraging in adults with the 5-year OS of 70%. A recent analysis of adult HL patients in Saudi Arabia has reported an OS of 91% [10]. Many factors have possibly contributed to the low survival of our HL patients. Firstly, the modality of the treatment used in our centre. The option of using a single modality therapy for treating HL by some oncology centres in the developing world [11], including ours, is not only because of unavailability or poor radiotherapy services. However, it is also because this modality has been considered by the NCCN guidelines as an alternative treatment option [37]. Taking into consideration the current survival results, oncologists and care providers in our facility should consider the use of alternative treatment options, possibly the combined chemo-radio treatment modality. In the ESMO clinical recommendation, consolidation radiotherapy is part of the treatment of patients with HL even in the early stages [38,39]. A systematic review analysis concluded that using combined chemo-radiotherapy improves tumour control and OS in the patients with HL, mainly in those with early stage disease [40].

Secondly, the interrupted therapy that some HL patients had received during their treatment course. Not all patients had regularly received the theoretical dosage of drugs because of unavailability of different drug items at the different times, which is regarded a week point in this analysis. Thirdly, the relatively short follow up period. The average follow up period in this study was 24 months; survival figures could have been more promising with longer follow up. Fourthly, the lack of effective salvage therapy. None of our HL patients had received brentuximab or had autologous bone marrow transplantation as they are not yet affordable in Iraq. Recent studies have shown that employing salvage therapy is highly effective for refractory or relapsed HL and improves patients’ survival [41].

In the current cohort, we investigated the relationship of a number of disease risk features and patients’ clinical characteristics such as age, gender, B-symptoms, advanced clinical stage, deep LNs, ESR >50 mm/hr, and LDH >500 IU/L to the 5-year OS and PFS rates. Generally, the associated risk features showed adverse correlation with the OS and PFS rates. However, only B-symptoms and high LDH revealed a significant correlation with the 5-year PFS rate. The absence of significant correlation between the survival and clinical stage of the disease is possibly due to the relatively short follow up period. Significant adverse correlation of the associated risk features with the survival had been reported by some studies but not by others [7,42–44].

In conclusion, the clinical and pathological characteristics of our HL patients followed the western developed pattern. Relapse was recorded in 20% of patients and death occurred in about 4%. The 5-year overall and progression-free survivals were far lower than the international rates, although the gap of difference was less in the paediatric age group. The associated risk features of the disease had a negative impact on survival rates; however, the associations did not reach statistically significant levels except for LDH.

## Supporting information

S1 File Hodgkin’s Lymphoma data file. (XLSX)
